# Information Regarding Dermatology as Seen on the Social Media Platform TikTok

**DOI:** 10.2196/59597

**Published:** 2025-01-28

**Authors:** Alexandra Rose Nigro, Alim Osman, Ryan Charles Saal, Robert James Smith

**Affiliations:** 1 Department of Dermatology, Eastern Virginia Medical School, 825 Fairfax Ave, Norfolk, VA, 23510, United States, 1 434-238-2324

**Keywords:** internet, social media, dermatology, TikTok, health care information, dermatological content, statistical analysis, dermatologists, online content, misinformation, engagement, health information, patient health, web platforms, patient education, digital health, online information, skin care, online videos

## Introduction

Americans spend an average of 2 hours and 7 minutes per day on social media [1]. It has become evident that patients are utilizing these networks to seek health care knowledge [2]. Previous studies have shown that dermatologists are using platforms such as Facebook or Instagram to disseminate dermatological content [3,4]. Interestingly, the information provided has often been inaccurate [5]. This study aims to describe the quantity and quality of dermatological content on TikTok, addressing the gap in research on dermatological information shared on this platform.

## Methods

### Overview

The TikTok platform was screened in November 2022 for the following keywords: dermatology, derm, skin, skin care, aesthetician, dermatologist, esthetician, derm physician assistant, dermatology physician assistant, derm PA, dermatology nurse, derm nurse, and derm nurse practitioner. Accounts containing ≥50% of dermatological content were included. An engagement score for these accounts was calculated by dividing the total number of likes by the total number of followers. The top 10 accounts were isolated based on engagement score, and their top 10 videos underwent further analysis using the DISCERN criteria (Table 1). DISCERN scores are calculated by assessing health information using a set of 16 standardized questions that evaluate factors like reliability, clarity, and overall quality of information presented. Each question is rated on a scale from 1 to 5, with higher scores indicating more reliable and high-quality content. These accounts were then grouped and analyzed based on occupation (Table 2). Two investigators (AO and ARN) independently scored the videos; differences in scoring were resolved by discussion between them. Kruskal-Wallis, Mann-Whitney *U*, and 2-tailed independent *t* tests were used to analyze the data. These statistical tests assessed the differences in content quality and engagement across occupations, providing insight into significant disparities without assuming normal data distribution.

**Table 1.** TikTok video data of the top 10 accounts. The average video length, views, likes, comments, and DISCERN score of the top 10 included accounts based on engagement score are displayed.

Account	Average video length (of the top 10 videos) (seconds), n	Average number of views (of the top 10 videos), n	Average likes (of the top 10 videos), n	Average comments (of the top 10 videos), n	Average DISCERN score (of the top 10 videos), n
Account 1	16.6	1,049,350	102,880	297.5	34.3
Account 2	15.2	1,409,470	107,408.6	672.2	34
Account 3	39	16,640,000	3,200,000	12,121.5	31.1
Account 4	23.2	3,088,690	176,179.4	1349.1	33.1
Account 5	20.5	2,660,000	122,340	480.8	34.8
Account 6^a^	50.8	64,920,000	4,187,020	20,217.6	32
Account 7^a^	44.7	13,590,000	121,703.5	623.4	37.6
Account 8^a^	21.5	26,970,000	1,115,094.8	6137.4	34.5
Account 9^a^	11	16,110,000	527,120	4983.3	32.3
Account 10^a^	16.7	8,120,000	721,210	2517.6	34.5

a Account belonging to a dermatologist.

**Table 2.** TikTok video data of the top 10 accounts. The analysis of the top 10 accounts from Table 1 based on occupation are displayed.

Account owner occupation	Engagement score, mean (SD)	Total videos, mean (SD)	Informational videos, mean (SD)	Number of videos with TikTok-verified paid sponsorship, mean (SD)
Dermatologists	21.66 (5.40)	795 (562.84)	691.22 (497.71)	18.22 (23.95)
Medical clinics	32.96 (14.54)	402.25 (243.48)	327 (274.68)	0 (0)
Aestheticians	26.47 (21.88)	478 (238.58)	316.5 (171.99)	3.17 (2.56)
Medical spas	15.77 (2.39)	279.5 (19.09)	199.5 (13.43)	0 (0)

### Ethical Considerations

This study was exempt from institutional review board approval, as only publicly available data were used.

## Results

Of the 22,411 TikTok videos analyzed, 17,085 (76.3%) videos were informational videos discussing skin and dermatology. Of these, 502 (2.2%) videos featured TikTok-verified paid sponsorships, with dermatologists comprising 328 (65%) videos of these. Among the 94 accounts analyzed, 35 met the inclusion criteria, including 18 (51.4%) dermatologists, 6 (17.1%) aestheticians, 4 (11.4%) medical clinics, and 2 (5.7%) medical spas. Using the Kruskal-Wallis test, significant differences in engagement scores were found across groups (*P*<.001), with medical clinics and aestheticians showing the highest average engagement scores.

A 2-tailed independent *t* test was used to confirm statistical significance between DISCERN scores of the dermatologist-run and nondermatologist-run accounts. Dermatologist-run accounts had significantly more views (25,942,000 vs 4,969,502; *P*<.001) and comments (6895.86 vs 2984.22; *P*=.04). Overall DISCERN scores did not differ between dermatologist-run and nondermatologist-run accounts (34.18 vs 33.46; *P*=.53), but subsections of the DISCERN scale did show significant differences. Dermatologist-run accounts were more likely to refer to areas of uncertainty (1.76 vs 1.22; *P*<.01) and describe how each treatment works (2 vs 1.48; *P*=.03). However, nondermatologist-run accounts were more likely to describe each treatment’s risks (1.56 vs 1.24; *P*=.049). The mentioned scores were still very low for both groups, suggesting an overall low quality of content for the videos.

## Discussion

There is a high volume of dermatological content produced and consumed on TikTok. Prior research demonstrates that consumer trust in TikTok content is high [6]. However, this study reveals that the most productive content creators in dermatology’s TikTok videos are of poor educational accuracy and quality. Physicians should be aware of the expanding role of social media as a source of medical information and possible misinformation. Dermatologists should consider if they have an obligation to produce unbiased, ethical, and accurate content. Social media platforms also bear a responsibility in regulating health-related content. Loeb et al [7] found widespread cancer misinformation on social media, often finding dissemination of unproven treatment options, demonstrating one example of how medical misinformation on social media can potentially harm patients. Though dermatologists should only report accurate information for proper patient care, it is equally important for social media platforms to at least label content as potential misinformation to prevent inadequate skin care.

Additionally, it is important to note the differences in the content reported between dermatologists and nondermatologists. With dermatologists more frequently referencing treatment uncertainty, viewers may perceive this as physicians practicing cautious, evidence-based medicine. With nondermatologists more frequently discussing risks, this may lead to consumers avoiding treatments and seeking alternative treatment solutions. Together, these differing emphases could shape consumer attitudes towards dermatological care, treatment choices, and even trust in professional advice versus influencer-driven content. Further exploration of these impacts is warranted to better understand the influence of social media on dermatological care.
